# Process-based therapy vs. routine-CBT for difficult-to-treat mood and anxiety disorders: study protocol for a randomized controlled trial

**DOI:** 10.1186/s13063-024-08689-3

**Published:** 2024-12-19

**Authors:** Ulrich Stangier, Viktoria Kohl, Nora Görg, Lucie Sendig, Bettina Hufschmidt, Desiree Bonarius, Arwin Nemani, Mareike Ebert, Stefan G. Hofmann

**Affiliations:** 1 Department of Clinical Psychology and Psychotherapy, Goethe University Frankfurt, Varrentrappstr. 40-42, Frankfurt, 60486 Germany; 2 Department of Psychology, Philipps University Marburg, Schulstr. 12, 35037 Marburg/Lahn, Germany

**Keywords:** Process-based therapy, Dynamic network analysis, Difficult to treat depression, Cognitive behavioral therapy, Randomized controlled trial

## Abstract

**Background:**

Process-based therapy (PBT) is a new framework to intervention planning, based on the use of ecological momentary assessment (EMA) data and dynamic and idiographic network analyses. Support for its applicability has been reported from a single-case studies. Here, we examine the feasibility and effectiveness of PBT in a larger clinical sample. We have translated a training manual of PBT and modified for delivery of CBT in mental health service. The aim of this study is to test the relative efficacy of PBT compared to traditional CBT delivered in routine practice (r-CBT) for difficult-to-treat mood and anxiety disorders.

**Methods:**

The study is a randomized controlled trial (RCT) of PBT vs r-CBT for difficult-to-treat unipolar depression and anxiety disorders. In total, 80 patients are recruited at an outpatient clinic and included in two intervention arms.

Primary outcome is emotional distress; secondary outcomes include psychological well-being and quality of life, adaptive behavior, psychological flexibility, and reflective functioning. Assessments of outcome variables are conducted before and after therapy and at 6 months follow-up. Weekly patient-rated outcomes are collected for every session to investigate process of change. Outcome assessors, blind to treatment allocation, will perform the observer-based symptom ratings, and adherence with manual will be monitored using self-report.

**Discussion:**

The current study will be the first RCT of PBT in a health care setting. The planned moderator and mediator analyses will clarify the mechanisms of change in psychotherapy and the association between personalized assessment based on dynamic network analysis and treatment effect.

**Trial registration:**

ClinicalTrials.gov NCT06517589. Registered 24 July 2024.

## Administrative information

**Table:** 

**Title {1}**	Process-based Therapy vs. routine-CBT for difficult-to-treat mood and anxiety disorders: Study protocol for a randomized controlled trial
Trial registration {2a and 2b}	Clinicaltrials.gov JWGUniversity record III L5-519/05.000.002. Registered 27 June 2024
Protocol version {3}	Research protocol, version 01, 10. August 2024
Funding {4}	This work is funded by the LOEWE Spitzenprofessur for Stefan G. Hofmann of the Hessian Ministry of Science and Arts
Author details {5a}	Ulrich Stangier, Viktoria Kohl, Nora Görg, Lucie Sendig, Bettina Hufschmidt, Desiree Bonarius, Arwin Nemani, Mareike Ebert: all Department of Clinical Psychology and Psychotherapy, Goethe University Frankfurt, Varrentrappstr. 40–42, 60,486 Frankfurt am Main, Germany Stefan G. Hofmann, Department of Psychology, Philipps University Marburg, Schulstr. 12, 35,037 Marburg/Lahn, Germany
Name and contact information for the trial sponsor {5b}	Prof. Dr. Ulrich Stangier, Varrentrappstr. 40/42, D-60486 Frankfurt; email: stangier@psych.uni-frankfurt.de
Role of sponsor {5c}	Ulrich Stangier: Principal Investigator of the trial. Stefan G. Hofmann: funder and Co-Principal Investigator. Study design, interpretation of data. Writing of the report. Decision to submit the report for publication Viktoria Kohl, Nora Görg, Lucie Sendig, Bettina Hufschmidt, Desiree Bonarius: trial management and data collection., Writing of the report Arwin Nemani, Mareike Ebert: analysis of data. Writing of the report

### Introduction

#### Background and rationale {6a}

Unipolar depression and anxiety disorders represent the most common mental health problems encountered in mental health care [1]. These conditions frequently lead to prolonged suffering, psychosocial impairment, and substantial socio-economic burden [2]. Thus, it is of major interest to optimize the efficacy of treatments in mental health services for these disorders.

The results of recent meta-analyses show that CBT is effective for both depression C[3] and anxiety disorders [4]. However, it has become evident that remission rates following CBT for depression and anxiety disorders are still disappointing [5], leading to a debate about the stagnating outcomes of CBT in depression [6] and anxiety disorders [7]. This highlights the importance of optimizing the efficacy of CBT.

One promising way to improve outcome is to personalize treatment by matching the specific mechanisms underlying psychological treatment to the psychological processes contributing to the psychological dysregulation of the individual patient. The process-based approach [8] focuses on this principle by gathering individual data through ecological momentary assessments that are used to develop idiographic dynamic networks to identify the key process maintaining the maladaptive pattern [9] and by choosing interventions which target change processes matching to this key process [10]. In contrast to a latent disease model, which assigns symptoms to an underlying disease, a disorder is seen as an individual network of interacting processes that is maladaptive in specific contexts [11]. However, in an extended evolutionary meta-model, context-bound psychological processes, instead of symptoms, are captured in an ideographic network on different psychological dimensions [12], and change processes are conceptualized according to the basic evolutionary principles of variation, selection, and retention [13]. Furthermore, in contrast to syndrome-based treatment packages, interventions are chosen according to the change processes which have been empirically supported in mediational analysis of RCTs [14].

Given the increased effort needed for the personalization, process-based therapy (PBT) appears especially relevant for patients who do not respond to empirically supported treatments. In psychiatric care, treatment-resistance is defined as a non-response to an adequate treatment given a correct diagnosis [15]. However, there is a large variety of definitions about the number and type of unsuccessful treatment attempts required to meet the criteria for treatment-resistance, in particular for treatment of depression [16]. To avoid the theoretical difficulties of categorially excluding any response to a treatment, it has been recently suggested to prefer the more comprehensive term ‘difficult-to-treat’ depression [16, 17]. In line with this recommendation, a Delphi method-based consensus on the definition of treatment-resistant anxiety disorders [18] proposed the term “difficult-to-treat” anxiety disorder due to its usefulness in the clinical context.

### Objectives {7}

The main objective is to investigate the effects of PBT compared with r-CBT for outpatients with difficult-to-treat unipolar depression or anxiety disorders. Primary outcome is emotional distress; secondary outcomes include psychological well-being and quality of life. Apart from the primary and secondary outcomes, we aim to investigate whether PBT, as proposed theoretically, produces changes in psychological flexibility, variation, selection, and retention of adaptive behavior, reflective functioning, and capacity for social and interpersonal pleasure. Furthermore, we will examine the possible mediating role of therapists’ adherence with the manual and patients’ changes in avoidance/behavioral control and cognitive style on outcome. Lastly, we will investigate potential moderators of outcome, i.e., patient characteristics including treatment expectations and experiential vs. rational learning style, as well as therapeutic alliance, across and within treatment conditions.

The primary outcome measure in this trial is the difference in symptoms of psychological distress between the two groups. We hypothesize that PBT leads to significantly greater reductions in symptoms of emotional distress, as compared to r-CBT after treatment and at 6-month follow-up. Furthermore, we expect that the secondary outcome measures positive mental health and quality of life are significantly more improved following PBT than r-CBT. Based on the treatment targets in PBT, we also hypothesize that variation, selection, and retention of adaptive behavior, psychological flexibility, and reflective functioning will be improved to a significantly higher degree following PBT compared with r-CBT. In addition, we hypothesize that changes in avoidance/behavioral control and cognitive style significantly mediate a positive outcome. Finally, we expect that a positive treatment expectations and an experiential learning style of the patients, as well as therapist’s adherence with the treatment manual, will significantly moderate treatment outcome.

### Trial design {8}

**Fig. 1:**
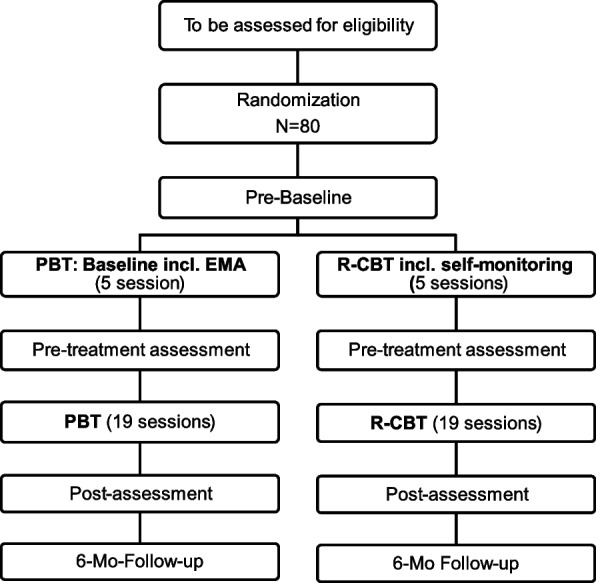
CONSORT flow diagram of the study design. EMA, ecological momentary assessment; PBT, process-based therapy; r-CBT, routine cognitive behavioral therapy

The current trial is an investigator-initiated, pragmatic, parallel group, two-arm superiority randomized clinical trial (RCT) with 1:1 allocation ratio. Two equally sized intervention arms, PBT and r-CBT, are compared. In total, we include 80 patients recruited from a university outpatient clinic. A CONSORT diagram is provided in Fig. 1.

## Methods: participants, interventions, and outcomes

### Study setting {9}

The study will be conducted within the outpatient clinic at the Department of Psychology, Goethe University Frankfurt.

Therapists are psychologists in advanced training for CBT, who are randomized to either PBT or r-CBT. Therapists randomized to PBT receive 20 h training focusing on personalization of treatment by

1. Deriving a hypothetical network model of the individual problem,
2. Guiding patients through the collection of EMA data,
3. Interpreting dynamic network models,
4. Drawing network-related treatment decisions, and.
5. Applying interventions based on mechanisms related to the central knot of the network.

Supervision of PBT treatments is offered by the first author. Therapists randomized to PBT receive a manual [19] containing essential procedures in network-based assessment and treatment.

Therapists randomized to r-CBT receive additional training in syndrome-based treatment selection and manualized treatments and their adaptation in complex cases.

In both conditions, therapists receive the same amount of supervision by qualified supervisors licensed for CBT.

### Eligibility criteria {10}

We aim to include 80 patients who meet the inclusion criteria: (1) a primary DSM-5 diagnosis of depressive or anxiety disorder, (2) at least two unsuccessful attempts of pharmacological or psychological treatment according to the German guidelines for the treatment of depression or anxiety disorders (Arbeitsgemeinschaft der Wissenschaftlichen Medizinischen Fachgesellschaften, AWMF), (3) age 18–65 years, (4) sufficient knowledge of the German language. Participating patients are not required to discontinue medication but to keep medication constant over the treatment period. Patients will be excluded in case of (1) increased suicidality, (2) substance abuse or dependency, (3) diagnose of borderline personality disorder, and (4) pervasive developmental disorder, psychotic disorder, eating disorder, bipolar disorder, or severe physical illness. Inclusion and exclusion criteria are assessed by intake clinicians using M.I.N.I. [20] adapted for DSM-5. Assessment of failure to respond to adequate treatment is based on an interview focusing on prior treatments and documents available from treating therapists or institutions.

### Who will take informed consent? {26a}

Potential trial participants are initially informed about the purpose of the trial by therapists at intake interview after registration at the outpatient clinic of the Department of Psychology. Patients who are interested in a participation receive detailed information from trial management staff about potential benefits and risks, their right to refuse participation and to withdraw consent at any time, and institutional affiliation of the trial at the university and sources of trial funding. Written consent is obtained according to the Declaration of Helsinki and as approved by the ethics committee of the Department of Psychology and Sports Sciences at Goethe University Frankfurt.

### Additional consent provisions for collection and use of participant data and biological specimens {26b}

N/A (no biological specimens are collected as part of this trial).

## Interventions

### Explanation for the choice of comparators {6b}

Clinical guidelines of the Association of the Scientific Medical Societies in Germany (Arbeitsgemeinschaft der Wissenschaftlichen Medizinischen Fachgesellschaften, AWMF) recommend the application of evidence-based treatment manuals of CBT as standard therapy for patients with anxiety disorders [60] and depression [61]. For patients with treatment failure in the past, no specific recommendations are given, except the adaptation of treatment to possible reasons for nonresponse or, if accepted by the patient, general change in treatment modality (from psychotherapy to pharmacotherapy or reverse). Thus, based on the guidelines cited above, standard interventions for patients with treatment failures do not differ from routine care and usually comprise traditional cognitive and behavioral interventions, but not third wave therapies.

### Intervention description {11a}

Both treatments comprise a 9-week baseline phase and a 20-week intervention phase.

#### Baseline phase

In the PBT condition, a hypothetical network model of the problem is derived, and EMA is conducted based on the key components of the model: situational context, cognition, emotion, body symptom, behavior, cognitive processing, and motivational schema. Focusing on these dimensions, typical examples of maladaptive responses are determined, and subsequently adaptive counterparts representing the desired poles of the dimensions that should be targeted in the treatment are defined. Then, participants are instructed to apply the mobile app Status (Vacay ©) which prompts the judgment of the seven dimensions established in the hypothetical network model on a bipolar scale ranging from − 100 (maladaptive) to + 100 (adaptive). Data are gathered in the situational context related to the problem. Therapists guide the adaptation of the items during the weekly sessions and help the patient to overcome difficulties in identifying situations and entering judgments of the components of the network model. EMA is completed when at least 100 measurements are taken. Then, a dynamic network analysis is computed for assessing autoregressive and cross-lagged effects of the variables [21]. These effects are estimated and visualized in a network representing variables as “nodes,” the effects between them as directed arrows (“edges”), and autoregressive effects as “self-loops.”

In the baseline phase of r-CBT, qualitative self-observational data are collected, using unstructured handwritten diaries of situations related to the problem, including notes on behaviors, cognitions, and emotions. Therapists assist patients in completing the diaries on a daily basis, support them to deepen the understanding of the problem, and derive a behavioral analysis of the individual problem based on the SORKC model according to Kanfer and Saslow (1965; [22]), as required for the application of CBT in German routine mental health care.

#### Treatment phase

This phase consists of 20 sessions for both treatment conditions. In PBT, treatment is initiated by a collaborative interpretation of the dynamic network model based on EMA collected during the baseline. Therapists are instructed to identify the central node, significant edges, self-loops, and positive or negative feedback loops between the nodes [19]. Based on the outcome of the dynamic network model, interventions are selected on the basis of empirical evidence for mechanisms of change matching to the central node of the individual patient, besides feedback loops and self-loops, as the key process maintaining the maladaptive pattern [10]. Interventions are conceptualized in the evolutionary framework as variation, selection, and retention of an adaptive mode of the central node related to the specific context of the problem [13]. The change of this variable is monitored using daily judgments on the basis of EMA. Further treatment planning focuses on additional targets to establish the adaptive modes of the dimensions as defined in the positive network model.

In r-CBT, as opposed to PBT, a naturalistic setting is retained for treatment decisions. Treatment planning follows traditional theories about the effects of the interventions on factors maintaining the disorder, e.g., avoidance and exposure in anxiety disorder or reduced reinforcement of activities and behavioral activation in depression [23]. Interventions are selected on the basis of common treatment manuals related to diagnoses, e.g., CBT for depression. Individual data from the behavioral analysis are used to tailor the techniques to the problem behaviors or dysfunctional thoughts of patients. Treatment process focuses mainly on the implementation of the manualized interventions adapted to the individual patient as recommended in the National guidelines for treatment of depression and anxiety disorders.

### Criteria for discontinuing or modifying allocated interventions {11b}

Adverse events will be monitored by the therapists, reviewed by the supervisors and reported to the PIs throughout the trial.

Study treatment must be terminated for one of the following reasons: (a) withdrawal of patient’s consent to study treatment or (b) study treatment termination by the investigator. Study treatment of a patient will be terminated by the investigator for one of the following reasons: (a) severe serious complications which makes it necessary to stop the study treatment, including acute suicidality, or (b) non-compliance with the study protocol. If the investigator terminates the treatment of the patient prematurely, he has to inform the patient about his decision, and the primary reason for withdrawal will be recorded in the patient file. If the patient caused the premature withdrawal, the data collected before termination may be used if the patient agrees and an informed consent for follow-up is signed by the patient. Allocated interventions will be adapted on consultation of the supervisor, in case of worsening of the patient’s condition.

### Strategies to improve adherence to interventions {11c}

Adherence to interventions will be supported by the strategies included in the treatment manuals. In r-CBT, these strategies include exploration of possible reasons for non-compliance, psychoeducation about the treatment, and clarification of treatment goals. In PBT, exploration of motivational factors, validation of barriers, and shared decision making in the selection of interventions will be applied.

### Relevant concomitant care permitted or prohibited during the trial {11d}

Concurrent and stabilized psychopharmacological treatment will be permitted if prescribed by a psychiatrist. Concurrent psychotherapy will be prohibited and leads to exclusion from the trial.

### Provisions for post-trial care {30}

If continuation of treatment is considered as necessary by the patient and the therapist at post-treatment, patients will be offered psychotherapy in the outpatient clinic.

### Outcomes {12}

#### Primary endpoints

Emotional distress, as assessed by the total score on the DASS-21 [25] after treatment, consisting of 20 sessions in both conditions, represents the primary outcome measure.

#### Secondary outcomes

Health-related quality of life will be assessed using the international standard uroqol-5D (EQ-5D; [26]). Positive mental health including emotional, psychological, and social aspects of well-being is assessed by the unidimensional Positive-Mental Health Scale (PMH, [27]) with 9 self-report items. The Anticipatory and Consummatory Interpersonal Pleasure Scale (ACIPS; [28]) is a 17-item self-report inventory and will be used to measure the pleasure patients experience in interpersonal situations. Psychological flexibility is measured by the 3-items self-report inventory Acceptance and Action Questionnaire Version 2 (AAQ-2; [29]).

The Reflective Functioning Questionnaire (RFQ; [30]) is a 54-item self-report inventory assessing the respondent’s ability to understand the internal mental states of self and others. Mentalizing capacity is divided in two subscales, Certainty and Uncertainty about mental states. The Client Satisfaction Questionnaire (CSQ-8; [31]) is an 8-item self-report instrument constructed to measure satisfaction with health services, which will be used only at post-assessment.

### Weekly and daily patients’ self-ratings of treatment process

The Processed Based Assessment Tool (PBAT; [32]) is a transtheoretical set of 18 self-report items, assessing variation, selection, and retention of adaptive behavior. The Depression Anxiety Stress Scale-10 (DASS-10; [33]) is the short version of the DASS-21, a self-report inventory measuring stress, anxiety, and depression in three subscales. The Cognitive-Behavioral-Therapy Skills Questionnaire (CBTSQ; [34]) is a 6-item self-report inventory measuring patients’ use of cognitive behavioral therapy interventions, with the dimensions behavioral activation and cognitive restructuring.

The Status-PBT (Vacay ©, 2024) is a mobile application for EMA during baseline phase which uses personalized questions and captures six dimensions (thought, emotion, body sensation, behavior, cognitive processing, motivational schema) on bipolar continuous scales ranging from − 100 to + 100. Similarity of the context with typical problem situations as defined in the hypothetical network model is rated on a continuous scale ranging from 0 to + 100.

### Therapists’ ratings

The Mini International Neuropsychiatric Interview [20] will be applied in the Version 7.0 for DSM-5 and contains 18 modules for the major mental disorders. At the enrolment interview, all modules will be used to assess inclusion criteria and comorbidity, but the modules anorexia and bulimia nervosa, binge eating, and prolonged grief are only carried out in full if there are indications in the interview. The presence of personality disorders is assessed according to the Structured Clinical Interview for DSM 5 for Personality Disorders (SCID-PD [35]).

A checklist of interventions for therapists is used after each session to check adherence, based on literature reviews [36, 37] and meta-analyses [38–40]. The Process-based Decision-making Questionnaire (PBDMQ) [24] contains 24 items and was developed to assess adherence with PBT. The “Clinical Global Impression Scale” (CGI; [41]) is 3-item observer-rated scale measuring the clinical impression of the severity of the mental disorder and is used to track changes in symptom severity.

### Moderators

The Kolb Learning Style Inventory [KLSI; [42]) will be administered at pre-treatment to assess active experimentation vs. abstract conceptualization learning style. The Credibility/Expectancy Questionnaire (CEQ; [43]) comprises 6 items three items of the subscale credibility and another three items of the expectancy subscale. The Helping Alliance Questionnaire (HAQ-11; [44]) comprises 11 items and measures therapeutic alliance. The questionnaire will be completed by patients and therapists at pre- and post-treatment.

### Participant timeline {13}

Patients are evaluated by clinicians to be eligible for participation in the study on the basis of inclusion and exclusion criteria described before. If patients are eligible for treatment, they will be provided with information about the project. Patients who consent to participate in the project will be included in the pretreatment assessment (see Table 1). Patients who cannot or will not participate in the study will be offered treatment as usual in the outpatient clinic.

**Table 1:** Overview of measurements

		**Inclusion**	**Baseline** (Sess. 1–9)	**Pre-treatment**	**Treatment** (Sess. 10–30)	**Post-treatment**	**Follow-up**
	Week instrument	0	1–9	10	11–31	32	57
**Therapist**	M.I.N.I/SCID-PD	**X**					
	CGI	**X**		**X**		**X**	**X**
	HAQ-11		**X** Week 7	**X**		**X**	
	FAMOS	**X**					
	Intervention checklist		**X** Week 9		**X** Weekly		
	PBDMQ		**X** Every 4 weeks		**X** Every 4 weeks		
**Patient**	DASS-21	**X**		**X**		**X**	**X**
	EQ-5D	**X**		**X**		**X**	**X**
	PMH	**X**		**X**		**X**	**X**
	ACIPS	**X**		**X**		**X**	**X**
	AAQ-II	**X**		**X**		**X**	**X**
	RFQ	**X**		**X**		**X**	**X**
	PBAT	**X**		**X**	**X** Weekly	**X**	**X**
	DASS-10				**X** Weekly		
	CBTSQ	**X**		**X**	**X** Weekly	**X**	**X**
	EMA/diary		**X** Daily weeks 5–9		**X** EMA: daily (central node)	**X** EMA: daily weeks 32–33	
	HAQ-11		**X** Week 7	**X**		**X**	
	KLSI			**X**			
	CEQ			**X**			
	CSQ					**X**	

*Abbreviations*: *M.I.N.I*., Mini-International Neuropsychiatric Interview; *SCID-PD*, Structured Clinical Interview for DSM 5 – Personality Disorders; *CGI*, Clinical Global Impression Scale; *HAQ-11*, Helping Alliance Questionnaire; *FAMOS*, Fragebogen zur Analyse Motivationaler Schemata, Kurzversion (Brief Assessment of Motivational schemata); *DASS-21*, Depression Anxiety Stress Scale 10 Items; *EQ-5D*, EuroQol 5 Dimensions; *PMH*, Positive Mental Health Scale; *ACIPS*, Anticipatory and Consummatory Interpersonal Pleasure Scale; *AAQ*, Acceptance and Action Questionnaire; *KLSI-12*, Kolb Learning Style Inventory; *CEQ*, Credibility Expectancy Questionnaire; *RFQ*, Reflective Functioning Questionnaire; *CSQ-8*, Client Satisfaction Questionnaire; *PBAT*, Process-Based Assessment Tool; *DASS-10*, Depression Anxiety Stress Scale 10 Items; *CBTSQ*, Cognitive Behavioral Therapy Skills Questionnaire; *PBDMQ*, Process-based Decision-making Questionnaire; *EMA*, Ecological Momentary Assessment

### Sample size {14}

Sample size estimation is based on the expected outcome in DASS-21, with treatment condition as between-subjects factor and a within-subjects repeated measurement factor (pre, post, follow-up). Since no previous trial has examined the effects of PBT, the expected effect size is set to a moderate effect of *f* = 0.25. Using an alpha level (*α*) of = 0.05 and a power of = 0.8 with correlation among repeated measures estimated at *r* = 0.4, 78 patients are needed (39 in each arm) to detect significant differences. To compensate for non-starters, a total sample of 80 patients will be randomized.

### Recruitment {15}

The outpatient clinic of the Department of Psychology has previously been engaged in several clinical trials. The number of attending patients is about 1000 patients per year. Thus, adequate participant enrolment can be expected to reach target sample size.

## Assignment of interventions: allocation

### Sequence generation {16a}

If eligibility for the study is confirmed, and informed consent to randomization is given, patient will be randomized. To allocate study participants to treatment conditions, a randomization list is created by the data management staff using the statistical software R.

### Concealment mechanism {16b}

The group allocations are printed out individually and placed in sealed envelopes.

### Implementation {16c}

For each newly included participant, a member of the trial management staff draws an envelope and reads off the group allocation. The same procedure is used to randomize therapists to the treatment conditions.

### Blinding

Outcome assessors will be blinded. No blinding of participants, care providers, or data analysts after randomization is planned.

### Who will be blinded {17a}

Outcome assessors will be blinded.

### Procedure for unblinding if needed {17b}

Not applicable.

### Data collection

#### Plans for assessment and collection of outcomes {18a}

An overview over assessments and collection of outcome during baseline, treatment, and follow-up period is given in Table 1, and the instruments are described in the “Outcomes {12}” section.

#### Plans to promote participant retention and complete follow-up {18b}

Participant retention during baseline, treatment, and follow-ups will be supported by the staff of the trial management. Participants who discontinue or deviate from intervention protocols will be requested to complete questionnaires at the measurement points according to the schedule presented in Table 1.

### Data management {19}

The trial will use electronic data collection and documentation, which is hosted by the company Vacay©. Entry of questionnaires will be performed by the participants. Coding, security, and storage of data will be ensured by the staff of the trial management. Access to the data is only allowed for persons who are documented as trial personnel and have received a training. The given data will be checked electronically for its plausibility and consistency in a multistage procedure. Detected inconsistencies and missing or implausible data will be clarified with queries (electronically or paper-based), and necessary changes will be carried out. At the end of trial, the database will be closed after data cleaning process. The pseudonymized patient data recorded are stored by Vacay in accordance with legal requirements.

#### Confidentiality {27}

Personal information about potential and enrolled participants will be pseudonymized and collected, shared, and maintained in accordance with the European data protection regulation.

### Plans for collection, laboratory evaluation, and storage of biological specimens for genetic or molecular analysis in this trial/future use {33}

Biological specimens are not obtained during the conduct of the trial.

## Statistical methods

### Statistical methods for primary and secondary outcomes {20a}

The primary outcome measure is the change in emotional distress from pretreatment to posttreatment, using the DASS-21 as primary outcome measure. Intention-to-treat analyses will be employed in this trial. For the statistical analysis of the primary outcome, we will use a repeated measures ANOVA with treatment condition as the between-subjects factor and time (pre-treatment and post-treatment) as the within-subjects factor. We will use a repeated measures MANOVA including a time treatment interaction to assess the effects of secondary outcomes, which are assumed to be moderately inter-correlated, followed by univariate repeated measures analyses.

For statistical analysis, the computer program SPSS ® (Statistical Package for Social Sciences, version 28) will be used.

#### Interim analyses {21b}

N/A (an interim analysis is not planned).

#### Methods for additional analyses{20b}

To examine potential mediator and moderators of treatment effects, structural equation modeling (SEM) and cross-lagged panel modeling (CLPM) will be employed using the R Package “lavaan” [45].

Dynamic network analysis of EMA data at baseline and at post-treatment will be computed using the R package “graphicalVAR,” with penalization parameters optimizing network structure [21, 46, 47] and detrending using the “forecast” R package [48]. Interpretation will focus on outstrength centrality to asses variable importance [49].

### Methods in analysis to handle protocol non-adherence and any statistical methods to handle missing data {20c}

Per-protocol analysis will include all participants attending at least 12 treatment sessions. For intention-to-treat analysis, missing data will be replaced, and multiple imputation by chained equations will be used, employing the “mice” R Package [50].

### Plans to give access to the full protocol, participant-level data, and statistical code {31c}

Individual participant data are intended to be shared after deidentification on a third-party website.

### Composition of the coordinating center and trial steering committee {5d}

Due to the small scale of our trial, no independent trial steering committee has been planned. The first author, U.S., is the principal investigator (PI) and will coordinate the trial.

### Composition of the data monitoring committee, its role and reporting structure {21a}

The study does not have a data monitoring committee.

### Adverse event reporting and harms {22}

All therapists involved in the trial are under continuous supervision by experienced, licensed clinicians. Spontaneously reported and solicited adverse events (e.g., worsening of symptoms or mental disorder) and serious adverse events (e.g., suicidality) will be collected by the therapists throughout the trial and reported in supervision. Serious adverse events related to the treatment will be reported immediately to the principal investigator. In an emergency, immediate contact with psychiatric inpatient facilities in the region can be established. Since the interventions will be carried out by clinical psychologists and will be supervised by an experienced therapist, any worsening of the mental health condition of the participants can be detected and met accordingly.

Somatic conditions are checked by physicians prior to the participation in the study. Risk of side effects is considered low, but potential treatment-related adverse events will be carefully monitored. Medication will be checked regularly by psychiatrists consulted by the participants in the trial who take psychopharmacological drugs. Patients participating in the trial are instructed to keep their medication and change only after consulting their psychiatrist.

Collected data on serious adverse events will be summarized by type and frequency, and regression analyses will be conducted to explore relations between adverse events and treatment.

### Frequency and plans for auditing trial conduct {23}

The conduct of the study will not be audited by an independent committee.

### Plans for communicating important protocol amendments to relevant parties {25}

Changes to the study protocol will be communicated to the ethics committee and the trial registry.

### Dissemination plans {31a}

The study protocol is made publicly available through this publication. The main results are intended to be published in a high-impact peer reviewed journal within 6 months after the trial end date. Individual participant data will be available for investigators whose proposed use of the data has been approved by an independent review committee identified for this purpose. Data will be available beginning 6 months and ending 36 months following article publication, after deidentification, and in accordance with the European General Data Protection Regulation (GDPR). To gain access, researchers requesting data will need to sign a data access agreement and contact the study sponsor.

## Discussion

The current study will be the first RCT investigating the evaluation of PBT in a mental health care setting. Since EMA is an important component of PBT that may contribute significantly to its effectiveness through reactivity to self-observation [51], we intend to disentangle the effects of intense self-monitoring before treatment from the effects of personalized interventions based on idiographic dynamic network analyses within treatment.

Analogue studies with students have shown that the collection of EMA data using the Status-PBT generates meaningful idiographic dynamic networks, depending on the quality of data collection [52–54]. There is a potential risk that the patients in PBT may not be able to recognize relevant dimensions of their problems in the initial sessions during baseline phase. Although therapists in the present trial are encouraged to adapt the definition of variables for EMA thoroughly within the initial sessions and guide the use of EMA, patients may not use the app after situations referring to the context of the problem and fail to cover relevant processes occurring in daily life. On the other hand, the capacity to perceive mental states may be increased through EMA [55]. Therefore, besides collecting qualitative data about the personal relevance of the results of dynamic network analyses presented to the patients, we also assess changes in mentalization as a possible effect of intense self-monitoring during the baseline.

The second important component of PBT is the personalization of treatment based on (a) information about the individual network of the problem and (b) matching interventions based on their effective components to the central node of the network [10]. This approach is opposed to r-CBT in the control condition, which uses treatment protocols based on diagnoses, consisting of fixed sets of treatment modules. Thus, PBT may improve the effectiveness of treatment by using empirical idiographic information of temporal and causal relationships between possible maintaining factors [14]. However, it should be noted that data used in PBT to determine if an intervention should be allocated are still based on nomothetic (i.e., group-based) research data based on diagnostic categories (e.g., for depression [39]). Further steps of the development of idionomic approaches to treatment decisions may include continuous dynamic network analyses of individual EMA data over the whole treatment, thus monitoring changes in the network and adapting interventions to them on different stages in the treatment [56]. A promising perspective may also be the application of control theory methods in the context of psychological dynamic networks [57–59], as they could provide a means to account for predicted intervention effects on an idiographic network; however, the application of such methods application in treatment studies requires further development. Despite these limitations, our study may provide a first proof of concept for the application of the process-based approach in a sample of patients with difficult-to-treat depressive and anxiety disorders. Using a randomized design and a manual for network-based assessment and algorithms for personalized treatment decisions, the current study will add to the preliminary results of single case studies [58], providing information on the efficacy of PBT as compared to r-CBT. The moderation and mediation analyses provide insight into the mechanisms of change and the relationship between treatment effects and patient characteristics. Future research may focus on the enhancement of PBT by developing personalized algorithms for treatment decisions on the basis of control theory, with special attention to patients who might not benefit from routine CBT in mental health care.

## Trial status

Research protocol, version 03, July 28, 2024. Training of therapists and outcome assessors is ongoing. Recruitment begins April 2024 and will be completed on 30 September 2025.

## Data Availability

The study protocol is made publicly available through this publication. The main results are intended to be published in a high-impact peer reviewed journal within 6 months after the trial end date (approximately 2026/27). Individual participant data will be available for investigators whose proposed use of the data has been approved by an independent review committee identified for this purpose. Data will be available beginning 6 months and ending 36 months following article publication.
